# Lymph Node Penetration From Gastric Burkitt Lymphoma in a Patient Living With HIV/AIDS

**DOI:** 10.7759/cureus.53905

**Published:** 2024-02-09

**Authors:** Ricardo José Razera, Ronaldo Modesto de Souza-Filho, Rosely Antunes Patzina, Jose C Ardengh, Richard Calanca

**Affiliations:** 1 Digestive Endoscopy, Instituto de Infectologia Emílio Ribas, São Paulo, BRA; 2 Pathology, Instituto de Infectologia Emílio Ribas, São Paulo, BRA; 3 Pathology, Universidade de São Paulo, São Paulo, BRA; 4 Gastrointestinal Endoscopy, Hospital das Clínicas de Ribeirão Preto, Ribeirão Preto, BRA; 5 Diagnóstico por Imagem, Universidade Federal de Sao Paulo, São Paulo, BRA

**Keywords:** immunohistochemistry and biopsy, upper endoscopy, acquired immune deficiency syndrome (aids), endemic burkitt’s lymphoma, primary non-hodgkin’s lymphoma

## Abstract

Non-Hodgkin's Lymphoma (NHL) in people living with Human Immunodeficiency Virus/Acquired Immunodeficiency Syndrome* (*HIV/AIDS) still constitutes a reality of high morbidity and mortality, sometimes not respecting the patient's degree of immunosuppression as it can even occur in those with a high CD4+ T lymphocyte count. Burkitt's lymphoma, in this sense, has been shown to be one of the main subtypes of this condition in this group of patients. This case report concerns a 32-year-old man diagnosed with metastatic gastric Burkitt's lymphoma after one month of his admission to a tertiary hospital for neurological complaints. The aim of this study is to raise an alert to suspect this diagnosis even in patients with adequate immunity, a well-done history, and a physical examination, which are the main pillars for reaching a diagnosis.

## Introduction

Non-Hodgkin's Lymphoma (NHL) in people living with Human Immunodeficiency Virus (HIV) and Acquired Immunodeficiency Syndrome (AIDS) (PLWHA) is responsible for a high prevalence and morbidity and mortality, accounting for 10% of cancers that affect PLWHA [1]. The systemic subtype associated with primary lymphoma of the central nervous system is part of the diseases that define AIDS as it characterizes advanced immunosuppression [2]. With the advent of antiretroviral therapy (ART), there was a progressive drop in the incidence of NHL in PLWHA despite the higher risk for those with a low CD4+ lymphocyte count and high viral load [3]. However, up to 25% of patients with CD4+ lymphocyte count above 500 cells/microliter may have Burkitt's lymphoma. The subject of the present study is to report patients who present with counts greater than 200 cells/microliter, especially young people requiring clinical suspicion in the face of conditions suggestive of the disease [4].

## Case presentation

**Figure 1:**
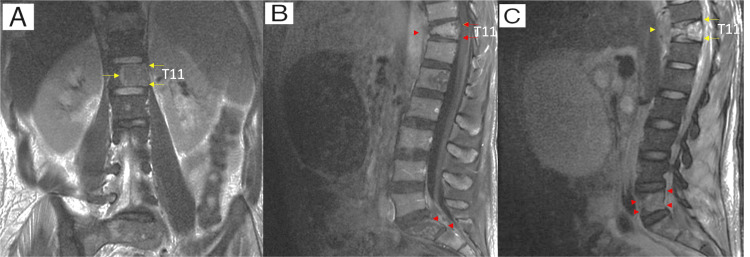
Neuroaxis MRI images Neuroaxis MRI showing multiple areas of osteolysis in the bone marrow of the T11 vertebral body (A and C: yellow arrows, B: red arrows); the sacral parts and the iliac bones (B and C: red arrows head). It is associated with a reduction in the height of the anterior aspect of the T11 vertebral body by more than 90% and a collection with peripheral contrast enhancement that extends to the pre-and paravertebral soft tissues at the T11 level (B and C: red and yellow arrows head). The imaging pattern is not specific but allows us to consider an association with the clinical suspicion of inflammatory/infectious involvement. Neoplastic nature is less likely, although it cannot be completely ruled out.

A 32-year-old man reported unquantified weight loss and generalized lymph node enlargement associated with hypoesthesia and lower limb paresis that began three months prior to admission and worsened within one week. The patient also presented a painless nodulation attached to the root of the left thigh, measuring approximately 4x3 cm. HIV was diagnosed upon admission, with tests showing a CD4 lymphocyte count of 99 and an HIV viral load of 65. Antiretroviral therapy (ART) was started with dolutegravir/lamivudine/tenofovir (TDF/3TC+DTG) combination. Neuroaxis MRI revealed areas of sacral and iliac bone osteolysis, with pathological fracture and T11 wedging, as well as a paravertebral collection with peripheral contrast enhancement, suggestive of infectious involvement, and the hypothesis of bone tuberculosis, was questioned (Figure 1).

**Figure 2:**
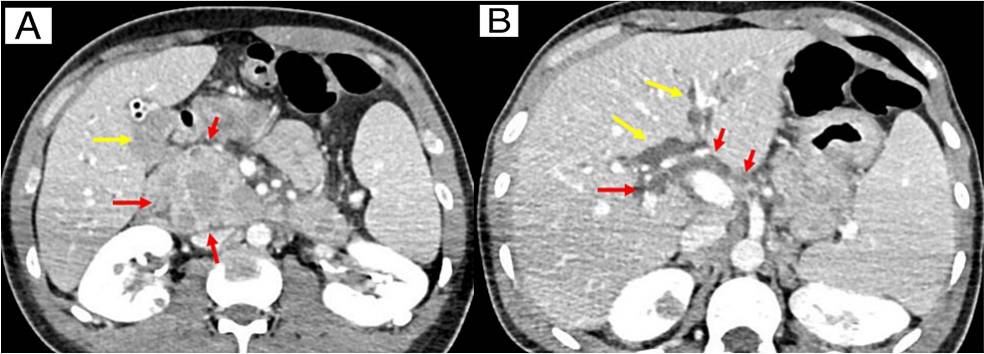
Abdominal CT scan images (axial view) (A) The image shows dilation of the bile ducts (yellow arrows), probably due to compression, which may correspond to granulomatous diseases (red arrows), such as disseminated tuberculosis or neoplastic dissemination of Burkitt's lymphoma. (B) The image shows multiple peripancreatic necrotic lymph node enlargements in the hepatic hilum (red arrows), exerting a compressive effect on the bile ducts.

Sputum investigation was carried out with negative regarding real-time polymerase chain reaction (PCR) based rapid molecular assay and bacilloscopy. Even so, empirical treatment was chosen for disseminated tuberculosis, given the condition with bone involvement and generalized lymph node enlargement. The insistence on the diagnosis of bone tuberculosis was made due to the possibility of false negative results in the tests presented, taking into consideration that the patient lived in Brazil, a country where the disease is highly prevalent, presenting a CD4 count lower than 200, which is compatible with the extra-pulmonary forms of the disease. The clinical and radiological presentation were other factors that influenced this possibility of not being immediately discharged. It was also opted for a biopsy of the mass in the root of the left thigh. Twenty days after admission the patient developed acute cholangitis, with an abdominal CT showing extrinsic compression of the bile duct from the Vater papilla (Figure 2).

**Figure 3:**
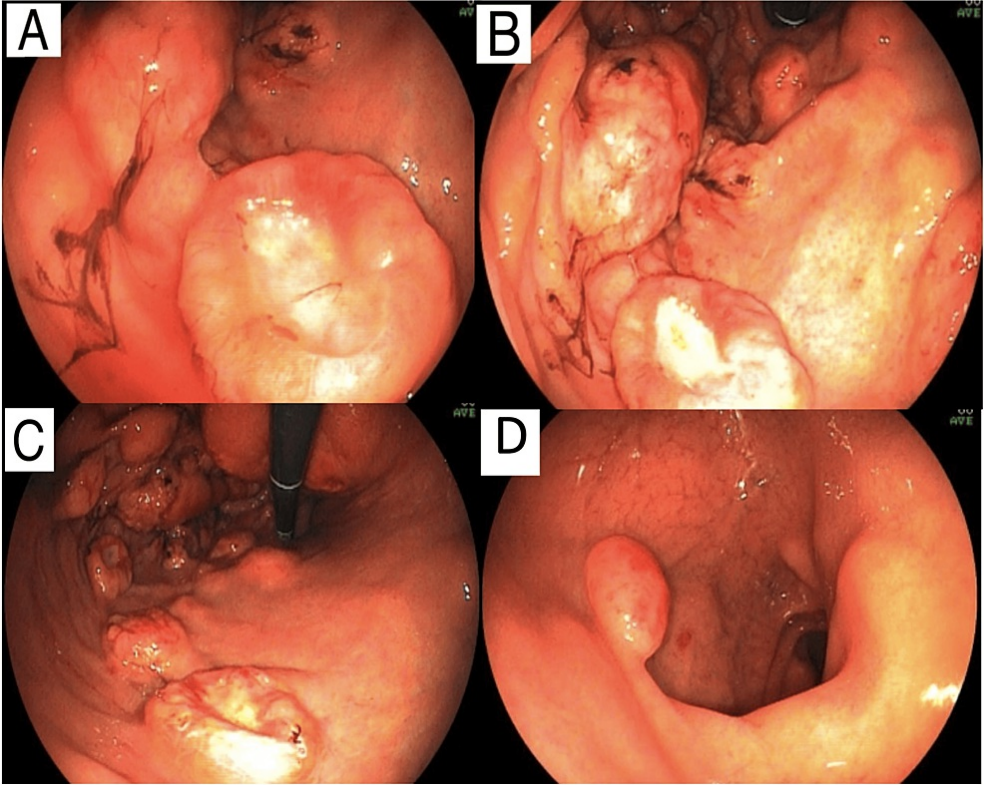
Upper gastrointestinal endoscopy images Upper GI showing, in the stomach, numerous elevated lesions, some with central ulceration, measuring between 10 and 25 mm, some of them friable, the largest located at the bottom, where there was still a bulge measuring around 3 cm (B and C). Such findings, on macroscopy, are compatible with extrinsic compression or submucosal involvement of findings compatible with lymphoproliferative or infectious disease, such as lymphomas, Kaposi's sarcoma or disseminated tuberculosis. Angular notch and antrum display flat-elevated lesions, reddish to burgundy, measuring between 6 and 10 mm (A). The duodenal bulb (D) shows two elevated lesions, measuring between 10 and 13 mm, with reddish surfaces located on the anterior and posterior walls.

An Upper Gastrointestinal Endoscopy (EGD) was performed, which revealed numerous elevated lesions in the stomach, some with central ulceration and others bulging in the fundus, body, and antrum region. Two elevated reddish lesions were also seen in the duodenum, with biopsies of the gastric ulcer and duodenal nodulation being performed (Figure 3). The patient then underwent endoscopic ultrasound and endoscopic retrograde cholangiopancreatography for urgent drainage with the insertion of a plastic prosthesis in the bile duct, with a stable evolution after the procedure.

**Figure 4:**
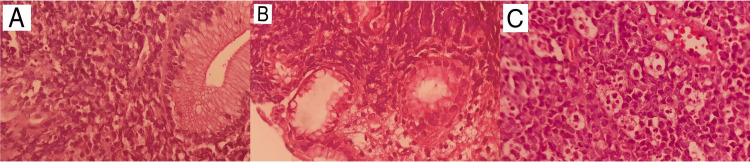
Histopathology images Magnification (H&E, 400X) showing atypical lymphoid cells surrounding and infiltrating duodenal (A) and gastric glands (B). Magnification shows sheets of monotonous intermediate-size cells with starry sky appearance in the inguinal lymph node (H&E, 400X) (C). Tingible body macrophages are phagocytic apoptotic debris.

**Figure 5:**
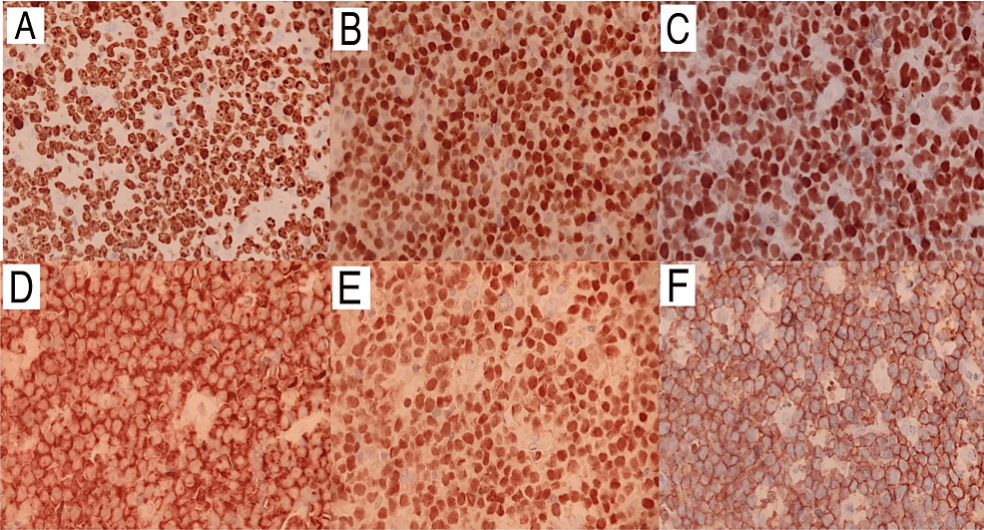
Immunohistochemistry images Immunohistochemistry images show tumor proliferation factor Ki67 approaching 100% (A), as well as positivity for markers cMYC (B), BCL6 (C), CD10 (D), PAX5 (E) and CD20 (F).

During hospitalization, biopsy results from findings in the gastrointestinal tract detected high-grade NHL (Figure 4). Likewise, a previously biopsied mass in the left lower limb showed the same pattern, with positive immunohistochemistry for CD20, CD10, BCL6, CMYC, and PAX5, but negative for BCL2, EBV, and TdT, with Ki67 of 100% (Figure 5) findings compatible with Burkitt's lymphoma. Chemotherapy with the cyclophosphamide, hydroxydaunorubicin, oncovin, and prednisone (CHOP) regimen was started, with progressive clinical improvement. The patient was then transferred to an oncology center for specific treatment, opting to continue treatment for tuberculosis until the conclusion of an investigation related to previously described bone lesions.

## Discussion

In approximately 70% of patients, the clinical picture of lymphomas in PLWHA is commonly composed of B-cell symptoms, including fever, weight loss, night sweats, and lymphadenopathy. Such signs and symptoms are confused with those of the immunocompetent population, with the differential diagnosis being established due to complaints related to frequent extranodal involvement of the disease in PLWHA [5-7], involving the gastrointestinal tract, liver, bone marrow, and central nervous system. Because they are nonspecific, these findings in several comorbidities can affect immunosuppressed individuals and become a diagnostic challenge. The case in question concerns Burkitt's lymphoma, an extra-nodal variant of systemic NHL classified as aggressive, ranking second (25%) in prevalence within the group of systemic NHL [8].

Involvement of the gastrointestinal tract is the main focus of the extra-nodal appearance of NHL. This fact draws attention to the fact that this disease forms part of the diagnostic hypotheses in the face of clinical syndromes similar to the one presented in the presented case due to its aggressiveness and high risk of complications, especially in PLWHA. The gastrointestinal tract is generally affected by secondary dissemination of lymphomas from a primary lymph node focus, with the stomach being the preferred organ, followed by the duodenum and anal/perianal region, as identified in our patient. The primary presentation is rare, making it difficult to establish the site of primary involvement of the disease [9].

Gastric Burkitt's lymphoma can mimic peptic ulcers, with dyspepsia and iron deficiency anemia, and can even progress to pyloric obstruction [10]. Histologically, it has monomorphic cells of medium size and a rounded nucleus, regular with dispersed chromatin, containing multiple small nucleoli. The “starry sky” pattern can be found due to the dispersion of macrophages with phagocytosed material. When it affects the intestine, the risk of obstruction increases, as does the risk of hemorrhage and perforation.

## Conclusions

In this case report, although the neurological symptoms attracted more attention initially, the symptoms also included lymph node enlargement and weight loss, which fit within the spectrum of complaints related to a lymphoproliferative neoplasm. The fact that the patient was investigated with upper gastrointestinal endoscopy in the face of a clinical complication arising from the GI highlights the importance of suspecting this disease so that the diagnosis can be established as early as possible so that the adoption of an appropriate therapeutic strategy can be adopted quickly.
